# Elucidation and valorization of the potent activity of *Commiphora myrrha* gum resin extract: antimicrobial and fibroblast wound healing activities

**DOI:** 10.1038/s41598-025-17079-x

**Published:** 2025-08-29

**Authors:** Rowena Mohamed Khalil, Nevine B. Ghanem, Heba Khairy

**Affiliations:** Department of Botany and Microbiology, Faculty of Science, Alexandria University, Moharam Bek, 21511 Alexandria, Egypt

**Keywords:** Green compounds, *Commiphora myrrha*, Benzofuran, Natural antimicrobial, Antioxidant, Wound healing, Antimicrobials, Bacteriology

## Abstract

This study investigated the antimicrobial, antioxidant, and wound-healing properties of *Commiphora myrrha* (*C. myrrha*) resin extract and its solvent fractions. Using ethanol, methanol, hexane, and water as extraction solvents, the ethanolic extract exhibited the strongest antimicrobial activity, which was attributed to its ability to solubilize a broad range of bioactive compounds. Further fractionation revealed that the ethyl acetate sub-fraction had the highest antimicrobial efficacy, including a 23 mm inhibition zone against *Klebsiella pneumoniae*. Scanning Electron Microscopy analysis provided novel insights into microbial cell disruption, showing significant morphological alterations in Gram-negative bacteria and *Candida albicans*, while *Staphylococcus aureus* exhibited minimal structural damage. The DPPH assay demonstrated potent antioxidant activity in the ethyl acetate fraction (IC₅₀: 0.92 mg/mL), indicating a strong radical-scavenging capacity. Solvent polarity was found to influence both antioxidant and antimicrobial activity, with ethyl acetate emerging as the optimal extraction solvent. The crude ethanolic extract showed moderate cytotoxicity toward human dermal fibroblasts (IC₅₀: 24.53 µg/mL) but promoted wound closure by 98.4% in vitro, suggesting its biocompatibility and regenerative potential. GC-MS profiling of the ethyl acetate fraction identified 70 compounds, including Benzofuran, Elemene, and Germacra-related sesquiterpenes, providing a chemical basis for the observed biological effects. This is the first study to comprehensively link the ethyl acetate fraction of *C. myrrha* to both antimicrobial and wound-healing activity, supporting its development as a multifunctional therapeutic agent. The results validate its traditional use and highlight its potential in modern pharmaceutical and nutraceutical applications.

**Supplementary Information:**

The online version contains supplementary material available at 10.1038/s41598-025-17079-x.

## Introduction

*Commiphora myrrha* (*C. myrrha*), a member of the Burseraceae family, is a resinous shrub native to arid regions of the Arabian Peninsula, East Africa, and the Mediterranean basin^1^. The resin of *Commiphora myrrha* (*myrrha*) is a thick, bitter-tasting, yellowish-brown gum with a pleasant aroma. This gum exudes as a fluid from resin ducts in the tree bark when the bark cracks naturally or is intentionally cut (tapping). Upon exposure to the air, this fluid hardens and turns into reddish-brown, irregular chunks called tears, which are then collected from the tree^2^. Myrrha has long been esteemed for its multifaceted pharmacological properties, particularly its wound-healing ability, antioxidant capacity in scavenging free radicals and mitigating oxidative stress, and broad-spectrum antimicrobial activity against pathogenic microorganisms^3^. The antimicrobial potential of *C. myrrha* has been extensively documented in both traditional and scientific literature^4^. Studies have demonstrated its effectiveness against a wide variety of microorganisms, such as bacteria, fungi, and protozoa^5^. Myrrha extracts and its essential oils exhibit inhibitory actions against clinically significant pathogens, including *Staphylococcus aureus*, *Pseudomonas aeruginosa*, *Klebsiella pneumoniae*, and *Candida albicans*^6^. These antimicrobial properties are attributed to the presence of bioactive compounds including sesquiterpenoids and phenolic acids which disrupt microbial cell membranes, inhibit enzymatic activity, and interfere with microbial proliferation^7^. In addition to its antimicrobial effects, *C. myrrha* contains a plethora of antioxidant compounds such as terpenoids, flavonoids, and phenolic acids which scavenge free radicals, chelate metal ions, and enhance endogenous antioxidant enzyme activity^8^. These antioxidative properties contribute to the maintenance of cellular homeostasis, the prevention of cellular damage, and the modulation of inflammatory responses^9^. Besides its antimicrobial and antioxidant effects *C. myrrha* extract promotes the proliferation of fibroblasts, enhances collagen deposition, and accelerates the epithelialization process, leading to faster wound closure^10^. A significant portion of the available drugs for wound management are not only expensive but also pose problems such as allergies and drug resistance. In contrast, phytomedicines for wound healing are not only inexpensive and affordable but are also safe^11^.

*Commiphora gileadensis* and *C. myrrha* are closely related species, both traditionally used for their therapeutic properties, including antimicrobial, anti-inflammatory, and wound-healing activities. While *C. gileadensis* has recently been studied using microwave-assisted extraction (MAE), leading to the identification of 30 novel phenolic compounds with strong antioxidant activity and high extraction efficiency, *C. myrrha* remains more widely known. *C. gileadensis* is a medicinal plant traditionally used for treating wounds, inflammation, and bacterial infections. Recent studies have demonstrated its rich phytochemical profile, particularly in phenolic and flavonoid compounds, which contribute to its antioxidant and therapeutic properties. A study utilizing microwave-assisted extraction (MAE) optimized the recovery of these bioactives from the plant’s bark, identifying 30 phenolic constituents for the first time and reporting strong in vitro antioxidant activity. These findings highlight the pharmaceutical potential of *C. gileadensis* and support the broader exploration of *Commiphora* species as sources of natural antimicrobial and wound-healing agents^12^.

Given the well-documented traditional and pharmacological significance of *C. myrrha* in antimicrobial, antioxidant, and wound-healing applications, this study aimed to comprehensively evaluate the bioactive potential of its crude ethanolic extract and solvent-partitioned fractions. Special emphasis was placed on identifying the most potent fraction and elucidating its mechanism of action against pathogenic microbes and in wound repair. Unlike most studies, which focus only on crude or essential oil extracts of *C. myrrha*, our work provides the first integrated evaluation of solvent-partitioned fractions, particularly the ethyl acetate sub-fraction. Considering the increasing demand for safer, plant-based therapeutic alternatives to address antibiotic resistance, oxidative stress-related disorders, and delayed wound healing, this study offers a timely and scientifically grounded investigation. The findings not only validate the traditional use of *C. myrrha* but also provide novel insights into its bioactive chemical constituents, particularly those concentrated in the ethyl acetate fraction, supporting its further development as a multifunctional agent for pharmaceutical and nutraceutical applications.

## Results

### *Commiphora myrrha* (*C. myrrha*) resin extraction and antimicrobial activity of the crude extract obtained using various solvents

The antimicrobial activity of the extracts obtained using different solvents was evaluated against a panel of microbial strains. Prior to testing with *C. myrrha* extracts, these strains were subjected to antimicrobial susceptibility screening using approximately 15 antibiotics and 6 antifungal agents. The purpose of this preliminary screening was to assess the resistance profile of the strains to conventional antimicrobial drugs. These results, summarized in Supplementary Figures S1 and S3, served as a reference point to evaluate the comparative efficacy of the plant extracts. Table 1 shows that the ethanolic extract exhibited superior antimicrobial efficacy, with an inhibition zone of up to 20.6 ± 1.24 mm against *Staphylococcus aureus* ATCC 29,213, exceeding that of the methanolic extract. Furthermore, ethanolic fraction showed a 23.6 ± 1.24 mm inhibition zone against *Candida albicans* ATCC 10,231. In contrast, the hexane and distilled water extracts showed no antimicrobial activity against any tested strains.

**Table 1:** Antimicrobial activity of *C. myrrha* resin extracts prepared using different solvents.

Tested microorganism	Zone of inhibition (mm)					
	Methanolic crude	Ethanolic crude	Ethanolic fractions			
			EtOAc	DCM	Hexane	Aqueous Layer
*Staphylococcus aureus* ATCC 29,213	16.3 ± 0.94^b^	20.6 ± 1.24^a^	17.6 ± 2.05^b^	6.6 ± 0.47^d^	11.0 ± 0.81^c^	13 ± 0.00^c^
*Klebsiella pneumoniae* ATCC 700,603	17.6 ± 0.47^bc^	18.3 ± 2.35^b^	23 ± 1.63^a^	11 ± 3.26^d^	10 ± 3.26^d^	17.6 ± 2.05^bc^
*Pseudomonas aeruginosa* ATCC 27,853	14.3 ± 0.94^c^	17.0 ± 1.63^b^	21.6 ± 1.69^a^	9.0 ± 2.16^d^	11 ± 0.81^cd^	15.3 ± 0.47^bc^
*Candida albicans* ATCC 10,231	19.3 ± 0.47^b^	23.6 ± 1.24^a^	20 ± 0.00^b^	0.0 ± 0.00^c^	0.0 ± 0.00^c^	16.3 ± 1.24^b^

The table includes data for methanolic and ethanolic extracts, as well as solvent fractions of the ethanolic extract (ethyl acetate [EtOAc], dichloromethane [DCM], hexane, and aqueous fractions). Values are the mean Inhibition zone (mm) ± SD (*n* = 3). Different letters within each organism indicate statistically significant differences between solvents according to one-way ANOVA followed by tukey’s HSD post hoc test (*p < 0.05*).

### Fractionation of ethanolic *C. myrrha* extract

The ethanolic fraction, which exhibited the largest inhibition zones, was selected for further fractionation to identify the most active component. This resulted in four sub-fractions: hexane, dichloromethane (DCM), ethyl acetate, and aqueous, all of which were evaluated for antimicrobial activity. The ethyl acetate fraction demonstrated the strongest effect, with a 23 ± 1.63 mm inhibition zone against *Klebsiella pneumoniae* ATCC 700,603, surpassing both the crude ethanolic extract and the other fractions (Table 1, Supplement Figure S2). Against *Candida albicans* ATCC 10,231, the ethyl acetate fraction produced a 20 ± 0 mm inhibition zone, slightly smaller than that of the crude extract. The aqueous fraction showed moderate inhibition zones against all tested strains. Consequently, the crude extract, the ethanolic fraction, and the aqueous fraction were preserved for further microbiological work.

### The minimum inhibitory concentration (MIC) and minimum bactericidal/fungicidal concentration (MBC/MFC)

The antimicrobial activity of the ethanolic extract, ethyl acetate fraction, and the aqueous fraction (included due to its antimicrobial effect in initial testing) was evaluated against a panel of microbial strains by determining their MICs and MBC/MFCs. The ethanolic extract exhibited MICs of 200 mg/mL against *Staphylococcus aureus* ATCC 29,213, *Klebsiella pneumoniae* ATCC 700,603, and *Pseudomonas aeruginosa* ATCC 27,853, with a slightly lower MIC of 180 mg/mL against *Candida albicans* ATCC 10,231. In comparison, the ethyl acetate fraction showed greater efficacy against *K. pneumoniae*, *P. aeruginosa*, and *C. albicans* (MICs of 180 mg/mL for each) compared to the crude ethanolic extract. However, *S. aureus* was less susceptible to the ethyl acetate fraction, displaying a higher MIC of 230 mg/mL. The MBCs of the ethanolic extract against *S. aureus*, *K. pneumoniae*, *P. aeruginosa*, and *C. albicans* were 204, 204, 232, and 202 mg/mL, respectively. The ethyl acetate fraction showed MBCs of 236, 202, 202, and 204 mg/mL against the same microorganisms (Table 2).

**Table 2:** Minimum inhibitory [etoac]oncentration (MIC) and minimum bactericidal/fungicidal [etoac]oncentration (MBC/MFC) of *C. myrrha* resin extracts prepared using different solvents.

Tested microorganism	MIC (mg/ mL)			MBC/MFC (mg/mL)		
	Ethanolic extract	EtOAc	Aqueous layer	Ethanolic extract	EtOAc	Aqueous layer
*Staphylococcus aureus* ATCC 29,213	200 ± 0.03^b^	230 ± 0.03^a^	230 ± 0.09^a^	204 ± 0.0^c^	236 ± 0.0^b^	238 ± 0.0^a^
*Klebsiella pneumoniae* ATCC 700,603	200 ± 0.03^a^	180 ± 0.05^b^	200 ± 0.04^a^	204 ± 0.0^a^	202 ± 0.0^b^	204 ± 0.0^a^
*Pseudomonas aeruginosa* ATCC 27,853	200 ± 0.02^a^	180 ± 0.04^b^	200 ± 0.09^a^	232 ± 0.0^b^	202 ± 0.0^c^	234 ± 0.0^a^
*Candida albicans* ATCC 10,231	180 ± 0.09^b^	180 ± 0.09^b^	200 ± 0.08^a^	202 ± 0.0^c^	204 ± 0.0^b^	232 ± 0.0^a^

The [etoac]able includes data for ethanolic extract, as well as solvent fractions of [etoac]he ethanolic extract (ethyl acetate [EtOAc] and aqueous fractions). Values are presented as mean ± sd (*n* = 3). Separate one-way [etoac]NOVA were performed for MIC and MBC/MFC within each organism, followed by tukey’s HSD post hoc [etoac]est. Different letters within each parameter indicate statistically difference between [etoac]reatments (*p < 0.05*).

### Cell morphology analyses using scanning electron microscope (SEM)

**Fig. 1:**
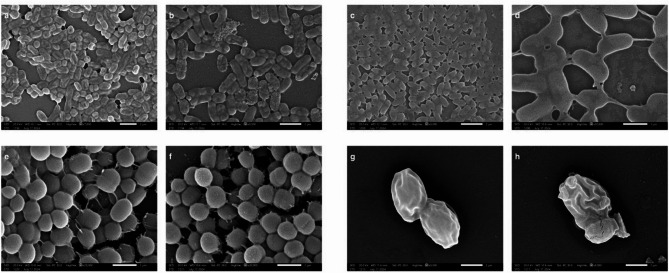
Scanning electron microscope of microorganisms treated with ethyl acetate fraction of *C. myrrha*. **a**: Untreated *K. pneumoniae* ATCC 700,603 cells (7000X); **b**: The treated *K. pneumoniae* ATCC 700,603 cells showing deformation of cells (9000X). **c**: Untreated *P. aeruginosa* ATCC 27,853 cells showed elongated rod cells (9000X). **d**: Treated *P. aeruginosa* ATCC 27,853 cells showed morphological changes (20000 and 9000X). **e**: Untreated *S. aureus* ATCC 29,213 cells were spherical (20000X); **f**: Treated *Staphylococcus aureus* ATCC 29,213 cells (20000X). **g**: Untreated *C. albicans* ATCC 10,231 cells exhibited typical oval morphology (9000X). **h**: Treated *C. albicans* ATCC 10,231 cells with deformation and cracks (9000X).

Scanning electron microscopy was used to examine the effect of the potent ethyl acetate fraction of *C. myrrha* resin on the cellular morphology of *Klebsiella pneumoniae* ATCC 700,603, *Pseudomonas aeruginosa* ATCC 27,853, *Staphylococcus aureus* ATCC 29,213, and *Candida albicans* ATCC 10,231 at their minimum inhibitory concentrations. The treatment induced noticeable morphological changes in the cells compared to untreated controls (Fig. 1, A–H).

The ethyl acetate fraction of *Commiphora myrrha* resin significantly altered the cellular morphology of Gram-negative bacteria (*Klebsiella pneumoniae* ATCC 700603 and *Pseudomonas aeruginosa* ATCC 27853) and the fungus *Candida albicans* at their minimum inhibitory concentrations, as observed by scanning electron microscopy (Fig. 1b, d and h). These changes included loss of the lipopolysaccharide layer and surface deformation in *K. pneumoniae*, irregular cell shapes and protrusions in *P. aeruginosa*, and cell wall cracks and deformation in *C. albicans*. In contrast, the Gram-positive bacterium *Staphylococcus aureus* showed no significant morphological changes upon treatment (Fig. 1f), consistent with its lower susceptibility to the extract in inhibition zone assays. These findings suggest that the *C. myrrha* resin extract compromises microbial viability by disrupting the integrity of the cell wall and interfering with essential cellular processes, particularly in Gram-negative bacteria and Candida strains. In Gram-negative bacteria such as *K. pneumoniae* and *P. aeruginosa*, SEM images revealed deformation of the outer membrane and loss of structural uniformity, likely due to damage to the lipopolysaccharide layer. In *C. albicans*, treated cells exhibited cracks and surface collapse, indicating that the extract may affect fungal cell wall biosynthesis and polysaccharide integrity. These morphological changes suggest that bioactive compounds within the extract interfere with the synthesis or stability of structural components such as peptidoglycan, chitin, or glucans, ultimately leading to compromised membrane function, leakage of cellular contents, and microbial death.

### Gas chromatography-mass spectrometry (GC-MS) analysis of the *C. myrrha* Ethyl acetate extract

The antimicrobial activity of the *C. myrrha* ethyl acetate extract indicates the presence of growth-inhibiting substances against the tested microorganisms. Gas Chromatography-Mass Spectrometry (GC-MS) analysis of this fraction identified 70 compounds, including tannins, sterols, steroids, isoprenoids (terpenoids), and several key bioactive constituents. Prominent among these were Benzofuran, 6-ethenyl-4,5,6,7-tetrahydro-3,6-dimethyl-5-isopropenyl, known for its antioxidant properties; Elemene, a sesquiterpene with anti-proliferative activity; various terpenes with antifungal potential; and Germacra-1(10),7,11-trien-15-oic acid, 8,12-epoxy-6-hydroxy, recognized for its antioxidant and antibacterial effects.

Additional compounds, such as gamma-lactone, germacrene B, and eremophilene, were also identified (Supplementary Table S1). Notably, Elemene, Germacra, germacrene B, and gamma-Muurolene were uniquely detected in the *C. myrrha* ethyl acetate fraction, whereas they were absent in the DCM or aqueous fractions.

### Biological impact

#### Evaluation of antioxidant activity

The DPPH radical-scavenging activity of *C. myrrha* resin crude ethanolic extract and its ethyl acetate (EtOAc), dichloromethane (DCM), hexane, and aqueous fractions was assessed. Using ascorbic acid as a reference standard (IC_50_ 0.04 mg/mL), the ethyl acetate fraction exhibited the strongest antioxidant potential (IC_50_ 0.92 mg/mL), followed by hexane (IC_50_ 1.06 mg/mL), crude ethanolic extract (IC_50_ 2.5 mg/mL), the aqueous fraction (IC_50_ 5.63 mg/mL), and the DCM fraction (IC_50_ 7.76 mg/mL). These results indicate that the DCM fraction exhibited the lowest scavenging activity, while the ethyl acetate fraction displayed the highest scavenging activity. Higher IC_50_ values indicate lower activity (Table 3).

**Table 3:** Antioxidant activity (% DPPH radical scavenging) of the crude ethanolic extract of *C. myrrha* resin and its fractions (ethyl acetate (EtOAc), dichloromethane (DCM), hexane, and aqueous layer).

C. myrrha fraction	DPPH-Scavenging %
Crude ethanolic extract	81.23 ± 0.72^b^
EtOAc fraction	90.75 ± 0.76^a^
Hexane fraction	89.40 ± 0.75^a^
Aqueous layer fraction	42.48 ± 0.59^c^
DCM fraction	22.36 ± 0.49^d^

Values are presented as mean ± sd (*n* = 3). One way ANOVA followed by tukey’s. HSD post hoc test was used to compare antioxidant activity among the different extracts. Different letters within each parameter indicate statistically difference between treatments (*p < 0.05*).

#### Cytotoxicity assessment of crude *C. myrrha* extract

**Fig. 2:**
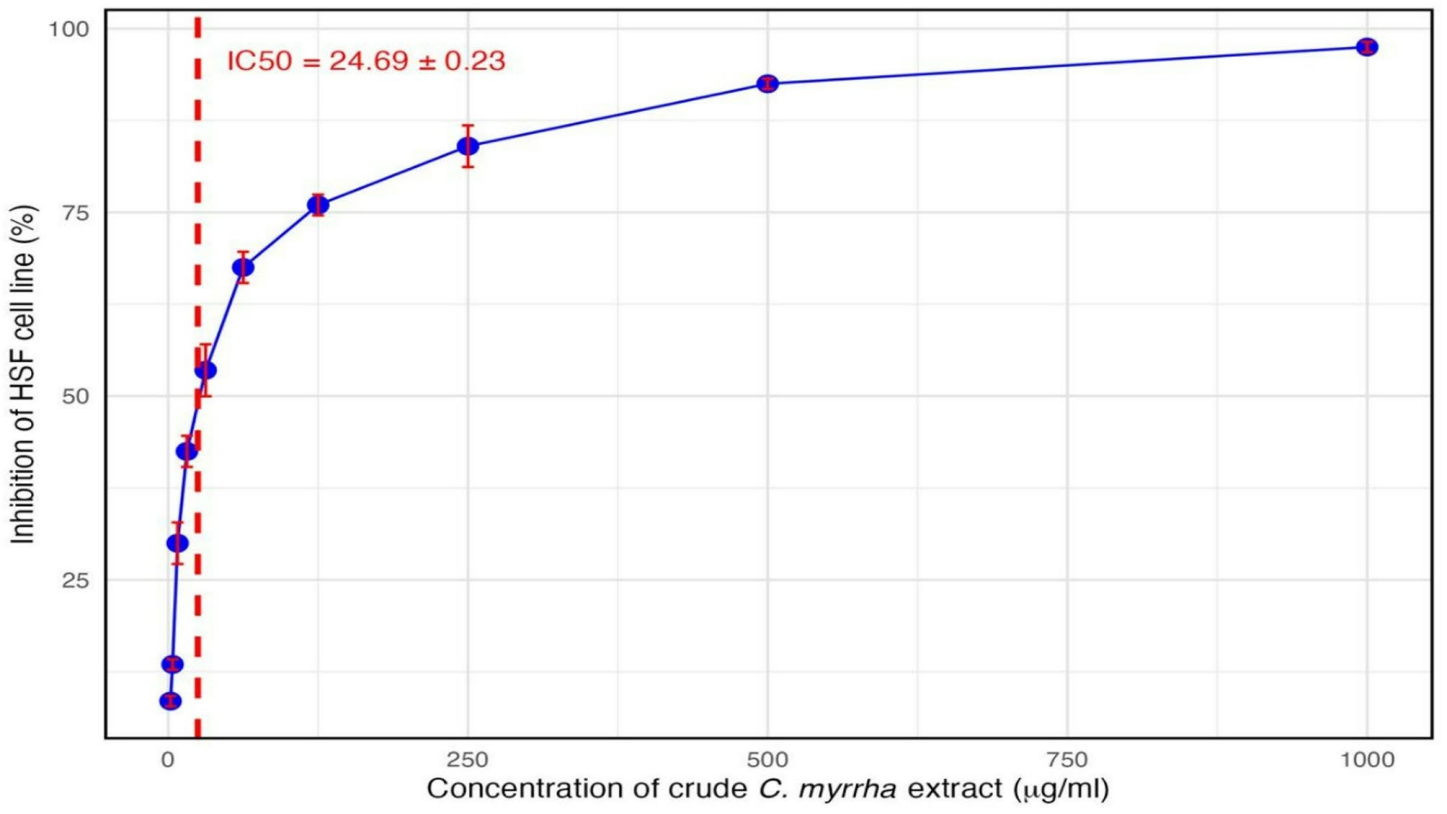
Cell Inhibition of crude *C. myrrha* ethanolic extract which illustrates the IC_50_ values with human dermal fibroblast cell line.

The cytotoxic effect of crude *C. myrrha* extract on normal human dermal fibroblast (HSF) cells was evaluated using the MTT assay across a broad concentration range (1.95 to 1000 µg/mL). As illustrated in Fig. 2, cell viability decreased in a dose-dependent manner with increasing extract concentrations. At the lowest concentration (1.95 µg/mL), minimal cytotoxicity was observed, with cell viability remaining above 90%, indicating good tolerability at low doses. A moderate reduction in viability was detected at intermediate concentrations (15.6–125 µg/mL), while a sharp decline occurred at higher doses. The highest concentration tested (1000 µg/mL) resulted in approximately 97% inhibition of cell viability, reflecting marked cytotoxicity. The calculated IC_50_ value was 24.53 µg/mL, representing the concentration at which 50% of cell viability was inhibited. This suggests that while the extract exhibits cytotoxic effects at higher concentrations, it remains relatively safe at lower levels. These findings support the use of crude myrrh extract at sub-cytotoxic concentrations—such as 100 µg/mL—for further biological assays, including wound-healing studies.

#### Wound healing assay of crude *C*. *myrrha* extract

**Fig. 3:**
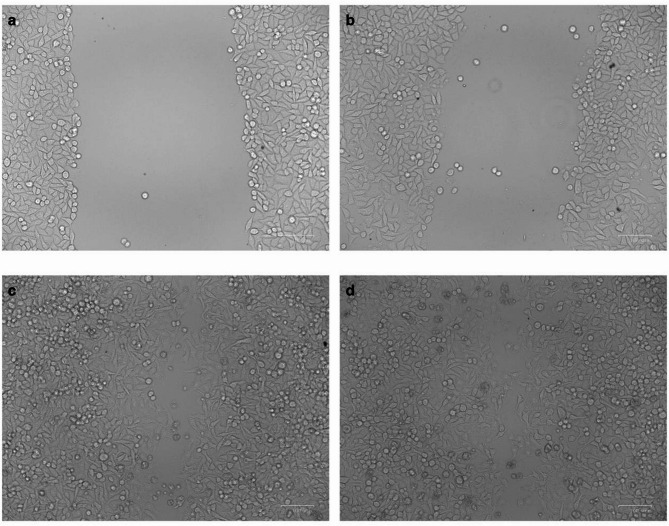
Scratch wound closure in human dermal fibroblast (HDF) cells under control and *Commiphora myrrha* crude ethanol extract treatment. Representative images at 0 h and 48 h showing: (a) control at 0 h, (b) treated at 0 h, (c) control after 48 h, and (d) treated after 48 h. Images were captured using a light microscope at 100× magnification.

**Fig. 4:**
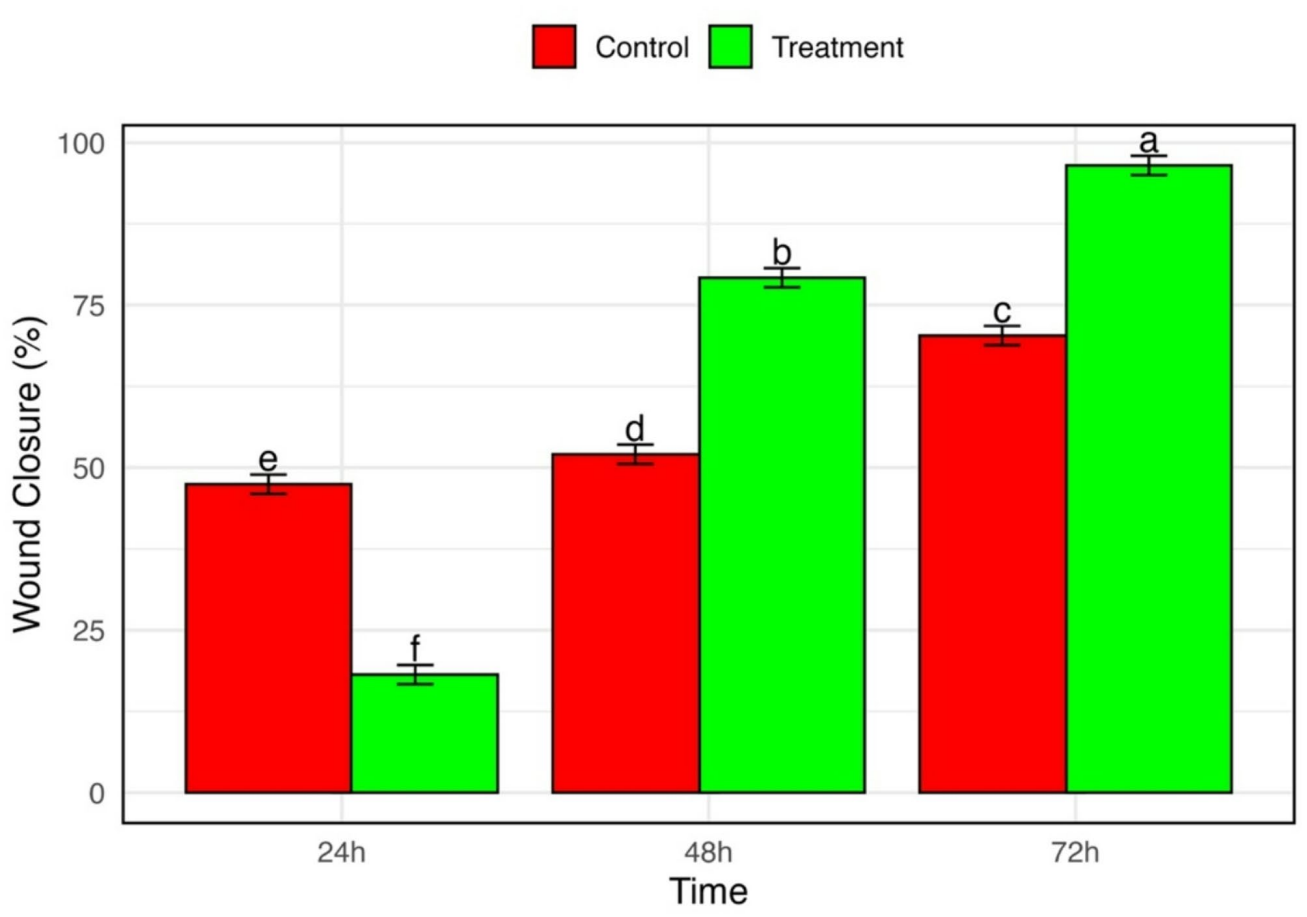
Quantitative analysis of scratch wound closure in human dermal fibroblast (HDF) cells treated with *Commiphora myrrha* crude ethanol extract. Bar graph shows the percentage of wound closure at different time points (0 h, 24 h, 48 h, 72 h) in control and treated groups. Data are presented as means ± standard deviation (SD), *n* = 3. Statistical groupings are indicated by letters (Tukey’s HSD, *p* < 0.05).

The wound-healing potential of crude myrrh extract (100 µg/mL) was evaluated using a scratch assay on human dermal fibroblast (HDF) cells. After 72 h of incubation, the treated group showed 98.4% wound closure, significantly higher than the 70.3% observed in the untreated control (Fig. 3). Two-way ANOVA revealed significant effects of Time, Treatment, and their interaction on wound closure percentage (*p* < 0.001). Tukey’s post hoc test confirmed a time-dependent increase in wound closure in both groups, with the treated group exhibiting significantly greater closure than the control at both 48 and 72 h (Fig. 4). These findings demonstrate that the crude myrrh extract significantly enhances HDF cell migration and promotes wound healing at the tested concentration.

## Discussion

Myrrha resin has been used for centuries in traditional Chinese and Indian medicine, frequently in conjunction with frankincense, to manage pain and inflammation related to trauma, arthritis, and fractures. It is also commonly employed in dermatological applications, demonstrating efficacy in treating skin ulcers and sores. Traditional practices have further documented its use in addressing tumors, enhancing blood circulation, and promoting tissue repair^13^. In the present study, the antimicrobial activity of *Commiphora myrrha* extracts was evaluated using various solvents: methanol, ethanol, hexane, and distilled water. Among these, the ethanolic extract exhibited the most potent antimicrobial activity. This observation aligns with previous findings that highlight ethanol as a particularly effective solvent for extracting bioactive antimicrobial compounds from *C. myrrha*. Ethanol-based extracts have demonstrated strong antimicrobial effects against pathogens such as *Klebsiella pneumoniae* ATCC 700,603, *Pseudomonas aeruginosa* ATCC 27,853, *Staphylococcus aureus* ATCC 29,213, and *Candida albicans* 10231^14^. The higher efficacy of the ethanolic extract may be attributed to ethanol’s ability to dissolve both polar and non-polar compounds, enabling the extraction of a wider spectrum of bioactive constituents, It has been documented that hydroethanolic solutions (85% ethanol) improve the solubilization of bioactive compounds like flavonoids, phenolics, and terpenoids compared to absolute ethanol, which can reduce the solubility of certain hydrophilic compounds^1,15^. In contrast, the methanolic, hexane, and aqueous extracts showed lower antimicrobial activity, likely due to the limited solubility of active compounds. Hexane, being non-polar, selectively extracts non-polar substances, which may not include the full range of antimicrobial agents present in *C. myrrha*^16^. Distilled water, a highly polar solvent, is similarly restricted in its ability to extract non-polar compounds, resulting in comparatively weaker antimicrobial effects^14^. These results underscore the critical role of solvent selection in optimizing the extraction of therapeutic compounds from medicinal plants. Moreover, MIC and MBC reported in this study were relatively high compared to standard synthetic antibiotics. It has been reported in the literature that plant-derived crude and semi-purified extracts often exhibit higher MIC values due to their complex mixture of bioactive and inactive constituents^17^. In evaluating the antimicrobial efficacy of *Commiphora myrrha* extracts, it is important to contextualize the observed minimum inhibitory concentration (MIC) values against standardized benchmarks. In our study, the MIC values for the crude ethanolic extract and its ethyl acetate fraction ranged from 180 to 230 mg/mL, depending on the microbial strain. While these results demonstrate measurable antimicrobial activity, they fall outside the conventional classifications of “high” (MIC ≤ 10 µg/mL) and “good” (MIC ≤ 100 µg/mL) antimicrobial efficacy, as defined in a recent meta-analysis of natural product antimicrobial screening^18^. For example, as reported by Qian et al., (2023)^18^, 48 out of 120 tested compounds with MICs ≤ 10 µg/mL were considered highly active, and the rest with MICs up to 100 µg/mL were rated as good candidates. In contrast, the higher MICs observed for *C. myrrha* extracts may be attributed to the use of crude or semi-purified fractions, which contain a complex mixture of both active and inactive constituents, diluting the effect of individual bioactive compounds. Despite these comparatively high MIC values, the extract still exhibited notable zone of inhibition activity in disc diffusion assays, suggesting that certain components may exert surface-level antimicrobial effects or that synergistic actions contribute to the observed activity. These findings underscore the potential of *C. myrrha* fractions as a source of bioactive agents, which may require further bio-guided fractionation and purification to isolate individual compounds with clinically relevant potency.

A comparative study was conducted on the antimicrobial activity of aqueous and ethanolic leaf extracts of *Commiphora wightii* and *Commiphora mukul*, prepared using Soxhlet extraction. The extracts were tested at varying concentrations against *Escherichia coli* and *Klebsiella pneumoniae*. Lower concentrations of both extracts did not produce significant zones of inhibition; however, at higher concentrations, both species exhibited measurable antibacterial activity. *C. wightii* ethanolic extract demonstrated the highest efficacy, with inhibition zones of 18 mm against *E. coli* and 20 mm against *K. pneumoniae*. In contrast, *C. mukul* extracts showed maximum zones of 6 mm and 15 mm, respectively. These findings suggest that *C. wightii* exhibits greater antimicrobial potential than *C. mukul*, particularly in its ethanolic form, and highlight interspecies variability within the *Commiphora* genus^19^.

Interestingly, *C. myrrha* extracts were more effective against Gram-negative bacteria than Gram-positive strains. This reduced susceptibility may be attributed to the unique structural features of *Staphylococcus aureus*. As a Gram-positive bacterium, *S. aureus* possesses a thicker peptidoglycan layer, which provides robust mechanical protection and structural rigidity. Unlike Gram-negative bacteria, it lacks an outer membrane, which in many cases acts as a selective permeability barrier—particularly to hydrophobic or amphipathic antimicrobial compounds. However, the thick and highly cross-linked peptidoglycan matrix in Gram-positive bacteria can also act as a physical barrier, reducing the diffusion and penetration of certain bioactive plant-derived molecules. Furthermore, Gram-positive cell walls often contain teichoic acids and other surface polymers that may bind or sequester antimicrobial agents, further limiting their access to intracellular targets^18,20^. These features could explain why *S. aureus* exhibited less pronounced morphological disruption and higher MIC values compared to the Gram-negative strains tested in this study. This aligned with the fact that the bacterial cell envelope, along with its structural components, represents an underexplored target for the development of novel antibacterial agents. Targeting the cell envelope offers a strategic advantage, as it may bypass or minimize the impact of well-characterized antimicrobial resistance (AMR) mechanisms, such as efflux pumps, enzymatic degradation, or target modification. By disrupting essential envelope structures—such as the peptidoglycan layer, membrane integrity, or biosynthesis pathways—new therapeutics can exert bactericidal effects through modes of action less susceptible to existing resistance pathways^18^. The most serious fungal infections are caused by *Candida albicans*, which is responsible for approximately 90% of documented cases. The antifungal properties exhibited by the *C. myrrha* extracts are in agreement with earlier research^21^, which associates their antimicrobial efficacy with the presence of volatile oils, terpenoids, phenolic compounds, flavonoids, and saponins. These substances are known to compromise fungal cell wall structure and mitochondrial integrity, ultimately disrupting energy metabolism^2^. To further isolate active compounds, the ethanolic extract underwent fractionation using hexane, dichloromethane (DCM), and ethyl acetate. Among these, the ethyl acetate fraction displayed greater antimicrobial activity against *K. pneumoniae* and *P. aeruginosa* than the crude ethanolic extract. This finding suggests the presence of specific bioactive compounds in the ethyl acetate fraction, particularly effective against Gram-negative bacteria. Ethyl acetate is known to extract various bioactive compounds such as glycosides, phenolic acids, flavonoids, amino acids, tannins, and semi-polar compounds like sesquiterpenoids (e.g., furanodiene, curzerene) and triterpenoids.

Conversely, the ethanolic extract was more effective against *S. aureus* and *C. albicans* compared to the ethyl acetate fraction^22^. This could be due to the broader range of antimicrobial compounds in the ethanolic extract, including terpenoids, phenolic acids, flavonoids, glycosides, fatty acids, and tannins^15^. Terpenoids are particularly noteworthy due to their broad-spectrum antibacterial, antifungal, and antiviral properties. They disrupt microbial membranes, leading to cellular leakage and death, while also exhibiting antioxidant effects that mitigate inflammation and oxidative stress^23^. Phenolic acids inhibit microbial growth by interfering with enzyme activity, DNA replication, and membrane function^24^. Flavonoids impair microbial enzyme systems, prevent biofilm formation, and compromise membrane integrity, while also protecting host tissues through their antioxidant activity^25^. Tannins exert antimicrobial effects by binding to microbial proteins and enzymes, inhibiting biofilm formation, and reducing oxidative stress^26^.

The antioxidant potential of *C. myrrha* extracts and their fractions was assessed using the DPPH (2,2-diphenyl-1-picrylhydrazyl) assay. The DCM fraction exhibited the highest DPPH value, indicating the lowest radical scavenging activity, while the ethyl acetate fraction showed a significantly lower DPPH value, reflecting stronger antioxidant activity. This pattern aligns with previous research, which suggests that less polar solvents like DCM extract fewer antioxidant compounds, whereas ethyl acetate effectively extracts phenolic acids and flavonoids known for their robust antioxidant properties^27^. The elevated antioxidant activity in the ethyl acetate fraction is likely due to the presence of sesquiterpenoids, diterpenes, triterpenes, and sterols. These compounds act as electron donors, neutralizing free radicals and interrupting radical chain reactions, thus playing a vital role in protecting cells from oxidative damage^28^.

Gas chromatography-mass spectroscopy (GC-MS) analysis further confirmed the chemical diversity of the ethanolic extract and its fractions. The ethyl acetate fraction, in particular, was rich in sesquiterpenes and flavonoids, compounds that are instrumental in the extract’s antimicrobial activity. These bioactive compounds interfere with microbial membrane integrity, enzyme function, biofilm development, and genetic replication, contributing to the broad-spectrum antimicrobial efficacy of *C. myrrha*^29^. These findings are consistent with earlier studies that identify sesquiterpenes and phenolic compounds as key bioactive components in *Commiphora* species^30,31^. The GC analysis thus provides valuable insights into the chemical basis underlying the biological activity of the various fractions^32,33^.

Among the compounds identified in the ethyl acetate fraction of *Commiphora myrrha*, several bioactive sesquiterpenes—including Elemene, Germacrene B, and γ-Muurolene—stand out for their well-documented pharmacological activities. Elemene, a bicyclic sesquiterpene, has been extensively studied for its broad-spectrum anticancer, antimicrobial, and anti-inflammatory properties, and is even in clinical use in some countries as part of adjunct cancer therapy. Germacrene B exhibits strong antibacterial and antifungal activity, and its lipophilic structure allows it to penetrate microbial membranes, contributing to cell wall disruption. γ-Muurolene, another abundant sesquiterpene hydrocarbon, has been reported to possess anti-inflammatory and antimicrobial properties, potentially enhancing both infection control and tissue repair^18,33^. The presence of these compounds supports the observed antimicrobial, antioxidant, and wound-healing activities of the *C. myrrha* extract and underscores its potential as a multifunctional natural therapeutic agent.

In addition to antimicrobial and antioxidant effects, the cytotoxicity and wound-healing potential of *C. myrrha* extracts were evaluated. The ethanolic extract demonstrated non-cytotoxic properties. To assess its wound healing potential, a cell scratch assay was performed on fibroblast cultures. Results showed that the extract promotes fibroblast migration a critical step in wound healing. The IC_50_ value of 24.53 µg/mL reflects the concentration at which 50% inhibition of cell viability occurs, the wound healing assay was conducted at a sub-cytotoxic concentration (10 µg/mL, i.e., 1/10th of IC_50_). At this level, the extract promoted fibroblast migration and wound closure without significant cytotoxicity, indicating a biphasic (hormetic) dose-response effect commonly observed in natural products. This dual activity suggests that the extract can support tissue regeneration at optimized, safe doses^33,34^. This effect is supported by prior studies indicating that *C. myrrha* enhances keratinocyte and fibroblast proliferation, collagen synthesis, angiogenesis, and tissue regeneration^34,35^. These wound-healing properties are largely attributed to bioactive terpenoids and phenolic acids, which modulate inflammatory cytokines and growth factors while reducing oxidative stress^36,37^. These results are highly relevant to DPPH activity of myrrha extracts, the antioxidant capacity is highly relevant to wound healing, as oxidative stress impairs tissue repair and promotes inflammation. The strong radical scavenging activity observed in the ethyl acetate fraction supports its potential to mitigate oxidative damage at wound sites, thereby complementing its regenerative effect. The DPPH assay thus provides a mechanistic basis for the extract’s wound-healing potential^38^. Recent studies on *Commiphora gileadensis* have demonstrated its rich phytochemical profile, particularly in phenolic and flavonoid compounds, which contribute to its antioxidant and therapeutic properties. A study utilizing microwave-assisted extraction (MAE) optimized the recovery of these bioactives from the plant’s bark, identifying 30 phenolic constituents for the first time and reporting strong in vitro antioxidant activity. These findings highlight the pharmaceutical potential of *C. gileadensis* and support the broader exploration of *Commiphora* species as sources of natural antimicrobial and wound-healing agents^12^.

Wound healing is a complex biological process that can be severely compromised by microbial colonization and infection. The selected organisms in our study—*Staphylococcus aureus*,* Pseudomonas aeruginosa*,* Klebsiella pneumoniae*, and *Candida albicans*—are clinically relevant pathogens frequently isolated from chronic wounds, burns, diabetic ulcers, and surgical site infections. These microorganisms are known to delay healing through biofilm formation, prolonged inflammation, and tissue degradation. For example *S. aureus* is a leading cause of skin and soft tissue infections, producing toxins that damage host tissue and evade immune clearance. While *P. aeruginosa* is commonly associated with burn wounds and exhibits strong biofilm-forming ability, which contributes to chronic infection and resistance to antibiotics. *K. pneumoniae* is increasingly reported in nosocomial wound infections, especially in immunocompromised patients, and often displays multidrug resistance. Besides, *C. albicans*, a fungal pathogen, frequently colonizes moist wound environments and can impair re-epithelialization, especially in diabetic and immunocompromised patients. Therefor the rational of this study give the rising incidence of polymicrobial and drug-resistant wound infections, there is a growing need for topical agents with broad-spectrum antimicrobial and wound-healing properties^18^. Our findings suggest that *C. myrrha* extract, particularly its ethyl acetate fraction, exhibits activity against both bacterial and fungal wound pathogens and demonstrates additional antioxidant and pro-regenerative effects, supporting its potential use as a natural wound care agent. This broader context reinforces the rationale for selecting these pathogens and supports further investigation o*f C. myrrha* extract in the development of topical formulations for infected or high-risk wounds.

In conclusion, *Commiphora myrrha* exhibits notable antimicrobial, antioxidant, and wound healing properties, attributed to a diverse array of bioactive compounds. Ethanol and ethyl acetate are particularly effective solvents for extracting these constituents, highlighting the importance of extraction methods in maximizing therapeutic potential. The fractions obtained were analyzed using GC-MS and further evaluated for antimicrobial, antioxidant, and wound-healing activities. To our knowledge, this is the first study to associate the ethyl acetate fraction of *C. myrrha* with both antimicrobial efficacy and fibroblast-driven wound closure, supported by SEM imaging and cytotoxicity assessment. These findings reinforce the traditional uses of *C. myrrha* and support its potential integration into modern medical and pharmaceutical applications. These promising results highlight the potential of *C. myrrha* resin for applications beyond traditional use, supporting its development into functional foods, such as capsules or pills, and other nutraceutical formulations.

## Methods

### *Commiphora myrrha* collection and crude extract preparation

Myrrha samples were obtained from traditional herbal markets in Alexandria, Egypt. Experimental research and field studies on plants (including the use of plant material) in this study complied with relevant institutional, national, and international guidelines and legislation. No wild collection was performed, and no endangered or protected species were used. As the material was commercially sourced, formal ethical approval was not required. Resinous *Commiphora myrrha* (100 g) was washed with distilled water and air-dried at 60 °C overnight before extraction with 85% ethanol, 100% methanol, 100% hexane, or distilled water. The desiccated powder was immersed in each solvent (ethanol, methanol, hexane, or distilled water) at 24 °C for 48 h. Filtrates were concentrated by rotary evaporation and dried at 24 °C after filtration through Whatman No. 1 paper. Extracts were dissolved in 2% dimethyl sulfoxide (DMSO) for further studies^39^.

### Antimicrobial activity

#### Microbial strains

Microbial isolates were provided from clinical hospitals in Alexandria, and their antimicrobial susceptibility to a panel of antibiotics and antifungal compounds were determined using the VITEK 2 system at Mabaret Alasafra, Alexandria as reported in supplementary Figure (S3). Based on their antibiotic resistance profiles, standard ATCC references were selected for further studies *Staphylococcus aureus* ATCC 29,213, *Pseudomonas aeruginosa* ATCC 27,853, *Klebsiella pneumoniae* ATCC 700,603, and *Candida albicans* ATCC 10,231.

#### Antimicrobial activity of extract obtained using various solvents

The antimicrobial activity of various *Commiphora myrrha* resin extracts (ethanolic, methanolic, hexane, and aqueous) was assessed using the Kirby-Bauer disc diffusion assay as per standard protocols^40^. This method was employed to evaluate the growth-inhibitory potential of the extracts against selected bacterial and fungal strains. The bacterial strains used in the assay included *Staphylococcus aureus* ATCC 29,213, *Pseudomonas aeruginosa* ATCC 27,853, and *Klebsiella pneumoniae* ATCC 700,603, while the fungal strain was *Candida albicans* ATCC 10,231. All strains were obtained from previously preserved cultures and sub-cultured on appropriate nutrient agar plates (Muller Hinton Agar for bacteria, Sabouraud Dextrose Agar for *C. albicans*) and incubated for 18–24 h prior to testing. Colonies from fresh cultures were suspended in sterile 0.85% saline and adjusted to a 0.5 McFarland turbidity standard, equivalent to approximately 1.5 × 10^8^ CFU/mL for bacterial strains and 1.0 × 10^6^ CFU/mL for yeast.

Sterile filter paper discs were impregnated with 100 µL of each extract solution at a concentration of 250 mg/mL, then allowed to dry at room temperature in a sterile environment to ensure uniform absorption. Dimethyl sulfoxide (2% DMSO) was used to dissolve the extracts and served as the negative control. In a separate experiment, standard antibiotic discs were used as positive controls for benchmarking purposes (supplement results). Plates were incubated at 30–37 °C for 18–24 h for bacteria, and at 30 °C for 24–48 h for *Candida albicans.* After incubation, the diameter of the inhibition zones around each disc was measured in millimeters (mm) using a digital caliper. The antimicrobial activity of each extract was determined by the size of the zone of inhibition. All experiments were conducted in triplicate, and results were reported as mean ± standard deviation (SD).

Based on the zone of inhibition data, the most active extracts (particularly the ethanolic extract) were further selected for detailed microbiological evaluation, including fractionation, MIC/MBC/MFC determination, and cell morphology analysis. The use of ATCC strains with clinical relevance ensured reproducibility and comparability with existing literature^39^.

#### **Fractionation and partitioning of the crude*C. myrrha*ethanolic extract**

The ethanolic extract with the highest antimicrobial activity was subjected to further fractionation by adding acetone, a polar solvent, mixing, and allowing it to stand for one hour followed by filtration through filter paper a process repeated twice. The resulting precipitate was collected, and the filtrate was evaporated under reduced pressure at 40 °C. The residue was then re-dissolved in 10% methanol and partitioned with hexane (non-polar solvent) using a separating funnel, resulting in the formation of two distinct layers, a hexane fraction (A) and an aqueous phase (B). The hexane fraction was separated, washed with distilled water, and evaporated under reduced pressure at 40 °C. The aqueous phase was then partitioned with dichloromethane (DCM, slightly polar solvent) to yield a DCM fraction (A) and an aqueous phase (B). The DCM fraction was collected, washed with distilled water, and evaporated under reduced pressure at 40 °C. The aqueous phase was further partitioned with ethyl acetate, a moderately polar solvent producing two layers: (A) the ethyl acetate fraction and (B) an aqueous phase. The ethyl acetate fraction was separated, washed with distilled water, and evaporated under reduced pressure. Finally, the remaining aqueous phase was evaporated at 40 °C to remove residual solvents and concentrate the extract^41^.

#### Antimicrobial activity assay of the ethanolic extract fractions

The disc diffusion technique was used to determine the antimicrobial efficacy of fractions resulting from the fractionation of *C. myrrha* resin extract. Muller Hinton/Sabouraud Dextrose agar plates were streaked with each bacterial and *Candida* strains, sterile filter paper discs were impregnated with 100 µl of the stock solution at 250 mg/mL of each of the following: crude *myrrha* resin, ethyl acetate fraction, dichloromethane (DCM) fraction, hexane fraction and aqueous layer fraction. Distilled water was used as a negative control. Plates were incubated at 30–37 °C for 18–24 h according to growth factors of each strain and at 30 °C for 24–48 h for *Candida albicans.* Inhibition zone diameters were measured, and all tests were conducted in triplicate. Standard deviations were calculated, and the most potent fraction was used for further experiments^39,41^.

#### Evaluation of the minimum inhibitory concentration (MIC) and minimum bactericidal/fungicidal concentration (MBC/MFC) of the active compound in myrrha extracts

The minimal inhibitory concentration (MIC) of the active compound in extracts was determined using the broth micro dilution technique. Briefly, stock solutions of crude myrrha extract, the ethyl acetate fraction, and the aqueous fraction were prepared at various concentrations (250, 230, 200, and 180 mg/mL) and added to 100 µL of microbial inoculum (10^8^ CFU/ml). The mixtures were incubated for 24 h at 37 °C^42^. The minimum bactericidal/fungicidal concentration (MBC/MFC) was determined by transferring 100 µL of broth from the MIC tubes showing zero or near-zero absorbance to fresh agar plates. MBC/MFC was confirmed by sub-culturing the test dilutions onto fresh solid medium and incubated for 24–48 h. The lowest dilution that yielded no bacterial/fungal growth on solid medium was recorded as MBC/MFC^39^.

#### Cell morphology analyses using scanning electron microscope (SEM)

The cell morphology of microorganisms treated with the potent *C. myrrha* fraction was examined using SEM. After a 24-hour incubation at the MIC of ethyl acetate fraction, the tubes were centrifuged to remove the supernatant. The cell pellets were then fixed, and the samples were subsequently dehydrated using alcohol. Samples were sputter-coated with gold and examined with scanning electron microscopy (JEOL JSM-IT200). All procedures were performed at the Scanning Electron Microscope Unit, Faculty of Science, Alexandria University, Egypt.

### Gas chromatography-mass spectroscopy (GC/MS)

GC/MS of the *Commiphora myrrha* resin extract (ethyl acetate fraction and DCM fraction) was carried out by utilizing a Shimadzu GCMS QP 2010 Ultra equipped with TRB-WAX column and TRB-5MS columns^43,44^.

### Biological impact

#### Antioxidant activity (DPPH free radical scavenging assay)

The 1,1-diphenyl-2-picryl-hydrazyl (DPPH) radical scavenging assay is commonly utilized for accurate quantification of the antioxidant activity of the compounds^45^. This technique is based on the ability of DPPH to decolorize in the presence of antioxidants. The DPPH free radical assay was performed using a previously established method^46^. Subsequently,100 µL of the test compounds at concentrations ranging from 0.2 to 25.6 mg/mL were mixed with 0.1 mL of the DPPH solution (0.2 mg/mL in ethanol). The absorbance was measured at 515 nm after a 30-minute incubation at room temperature. Ascorbic acid (Vitamin C), a well-known standard with strong antioxidant activity, was used as a positive control. The percentage of DPPH radical scavenging activity was calculated according to the following formula:

$${\text{DPPH~radical~scavenging~effect~}}\left( {\text{\% }} \right)=\frac{{{\text{Ac}} - {\text{As}}}}{{{\text{A~c}}}} \times 100$$

where: Ac = absorbance of the control (without sample), As = absorbance value of the standard or tested compound. The antioxidant ability of the sample was expressed as IC_50_ value^7^.

#### The cytotoxicity (MTT) of the crude extract of *C. myrrha*

Cell viability was assessed using the 3-(4, 5-dimethylthiazol-2-yl)-2,5 diphenyltetrazolium bromide (MTT) assay. The Human Dermal Fibroblast cell line (HDF) was obtained from the City of Scientific Research and Technological Applications (Alexandria, Egypt). 200 µL of HDF cell suspension that contained 3000 cells/ well was plated in 96-well plates and incubated for 24 h. After incubation, 100 µL of different concentrations of crude *C. myrrha* extract in RPMI medium without fetal bovine serum were added to the wells, and the plates were re-incubated for an additional 24 h in CO_2_ incubator (37 °C, 5% CO_2_, and 90% relative humidity). After 24 h of incubation, 20 µL of MTT solution was added to each well, and the plates were incubated for 3 h in the CO_2_ incubator to enhance the MTT to be reacted. Subsequently, the plates were centrifuged at 1650 rpm for 10 min, and the medium was carefully removed. The resulting formazan crystals (MTT byproduct) were re-suspended in 100 µL of DMSO, and absorbance was measured at 570 nm using an Optima spectrophotometer to determine safe doses exhibiting 100% cell viability^47^.

$${\text{Cell viability \% }}=\frac{{{\text{AT}} - {\text{Ab}}}}{{{\text{Ac}} - {\text{Ab}}}}{\text{~}} \times {\text{~}}100$$

A_T_ = absorbance of cells treated with different concentration of crude *C. myrrha* extract. A_C_ = absorbance of control untreated cells with culture medium only. A_b_ = absorbance of cells treated with vehicle of crude *C. myrrha* extract (RPMI without fetal bovine serum).

The cytotoxicity assay of the compound was expressed as IC_50_ and was calculated by the Graphpad Instat software using the % of viability calculated from the serial dilutions of each crude *C. myrrha* extract.

#### Wound healing of crude *C. myrrha* extract

The wound healing assay was performed on the human dermal fibroblast cell line (HDF), obtained from the City of Scientific Research and Technological Applications (Alexandria, Egypt). HDF cells were plated and incubated for 24 h in a CO_2_ incubator (37 °C, 5% CO_2,_ and 90% relative humidity). The following day, the culture medium was replaced with serum-free MEM to wash the cell monolayer, and then horizontal scratches were introduced into the confluent monolayer. The plate was thoroughly washed with phosphate-buffered saline (PBS). Control wells received a fresh medium, while test wells were treated with fresh medium containing crude *C. myrrha* extract at 1/10 of the IC_50_ value. The plate was then incubated for an additional 72 h. Migrating cells into the denuded zone were monitored and photographed using phase-contrast microscopy. The relative wound size was quantified using ImageJ software version 1.49o^48^.

### Statistical analysis

A one-way Analysis of Variance (ANOVA) was conducted to assess significant differences in the mean values of the zone of inhibition, minimum inhibitory concentration (MIC), and minimum bactericidal/fungicidal concentration (MBC/MFC) among different solvent extracts. The analysis was performed separately for each tested microorganism and for each parameter (zone of inhibition, MIC, and MBC/MFC) to evaluate the effects of the solvents: methanolic crude, ethanolic crude, ethanolic fractions (ethyl acetate [EtOAc], dichloromethane [DCM], hexane, and aqueous layer).

One-way ANOVA was also used to evaluate differences in DPPH radical scavenging activity (% inhibition) across the same set of extracts.

For the wound healing assay, a two-way ANOVA was applied to analyze the effects of treatment (control vs. *Commiphora myrrha* crude ethanol extract) and time (24 h, 48 h, 72 h) on the percentage of wound closure in human dermal fibroblast (HDF) cells. The 0 h time point was used as a reference for imaging but was excluded from statistical comparisons. When significant differences were detected (*p* < 0.05), Tukey’s Honestly Significant Difference (HSD) post hoc test was used for pairwise comparisons. Groups with significantly different means were labeled with distinct letters in the graphs.

All data are presented as mean ± standard deviation (SD) from three independent biological replicates (*n* = 3). Statistical analyses were conducted in R software (version 2025.05.0 Build 496) with a significance level of α = 0.05.

## Supplementary Information

Below is the link to the electronic supplementary material.

Supplementary Material 1

## Data Availability

No datasets were generated or analysed during the current study.
