# Supplementary material for: Elucidation and valorization of the potent activity of Commiphora myrrha gum resin extract: antimicrobial and fibroblast wound healing activities
Source: Sci Rep. 2025 Aug 29;15:31839. doi: 10.1038/s41598-025-17079-x (PMC12397419; doi:10.1038/s41598-025-17079-x)
Supplement: Supplementary file 1 — Supplementary Material 1 [file 41598_2025_17079_MOESM1_ESM.pdf]

Supplementary Information

Elucidation and valorization of the potent activity of *Commiphora myrrha* gum resin extract: Antimicrobial and fibroblast wound healing activities

Rowena Mohamed Khalil[1], Nevine B. Ghanem[1] and Heba Khairy[1*]

[1]Department of Botany and Microbiology, Faculty of Science, Alexandria University, [*]Alexandria, Egypt, To whom correspondence should be addressed: **Heba Khairy, email: heba.khairy@alexu.edu.eg**

Nevine Ghanem: ORCID: https://orcid.org/0000-0002-0441-0010

Heba Khairy: ORCID: https://orcid.org/0000-0001-6072-8202

Rowena Mohamed Khalil: ORCID: https://orcid.org/0009-0006-1147-5601

Address: Department of Botany and Microbiology, Faculty of Science, Alexandria University, Moharam Bek 21511, Alexandria, Egypt

Postal code: 21511

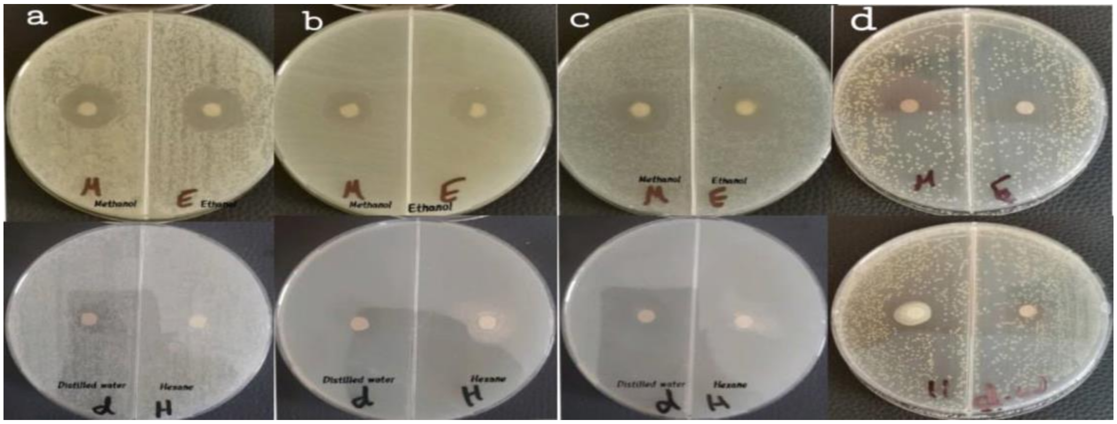

Figure (S1): Inhibition zones of methanolic (M), ethanolic (E), hexane (H) and distilled water (d) of *Commiphora myrrha* extract aganist (**a**) *Klebsiella pneumoniae*, (**b**) *Pseudomonas aeruginosa*, (**c**) *Staphylococcus aureus* and (**d**) *Candida albicans*

Note: All microbial cultures were standardized to a 0.5 McFarland turbidity standard (~1.5 × 10^8 CFU/mL) prior to inoculation to ensure consistency across experiments.

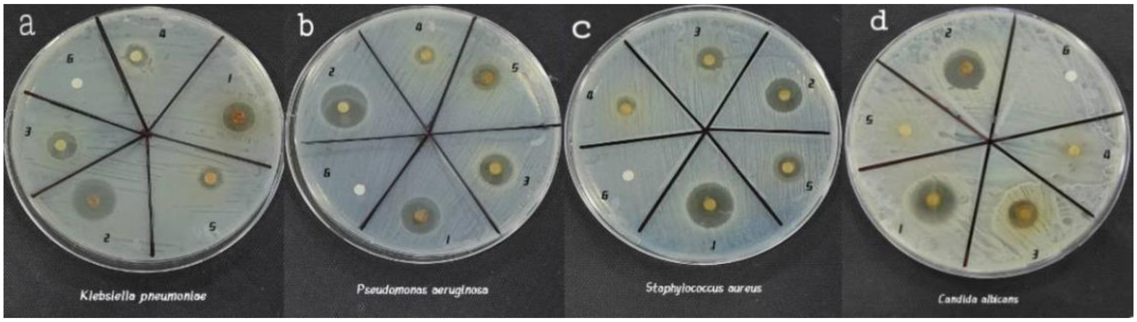

Figure (S2): Inhibition zones of crude ethanolic *Commiphora myrrha* extract and its fractions with **(1)** Crude ethanolic extract, **(2)** Ethyl acetate fraction, **(3)** Aqueous layer, **(4)** DCM fraction, **(5)** Hexane fraction and **(6)** Negative control against **(a)** *Klebsiella pneumoniae*, **(b)** *Pseudomonas aeruginosa*, **(c)** *Staphylococcus aureus* and **(d)** *Candida albicans*

Note: All microbial cultures were standardized to a 0.5 McFarland turbidity standard (~1.5 × 10^8 CFU/mL) prior to inoculation to ensure consistency across experiments.

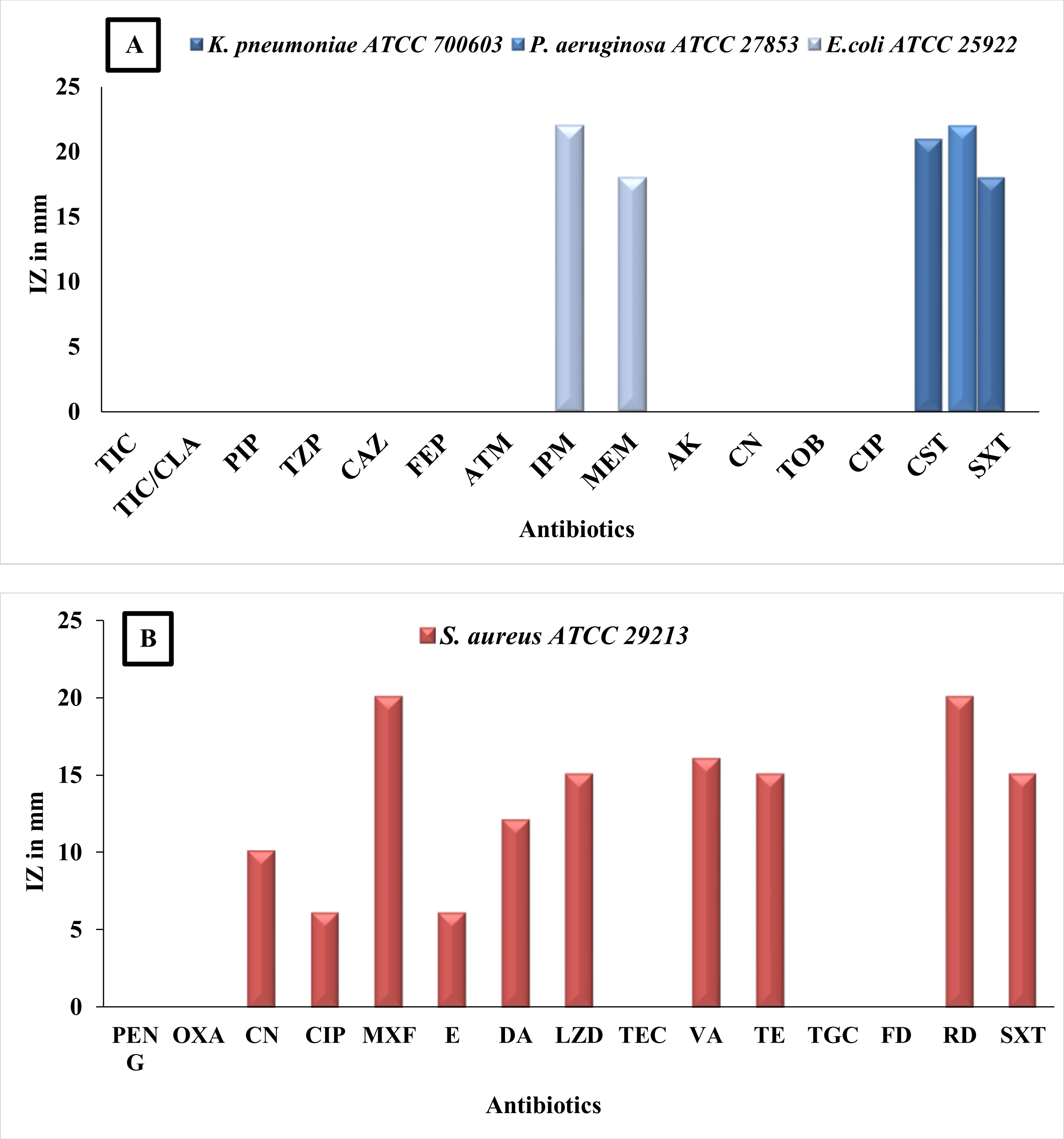
A
K. pneumoniae ATCC 700603
P. aeruginosa ATCC 27853
E.coli ATCC 25922
IZ in mm
0
5
10
15
20
25
TIC
TIC/CLA
PIP
TZP
CAZ
FEP
ATM
IPM
MEM
AK
CN
TOB
CIP
CST
SXT
Antibiotics
B
S. aureus ATCC 29213
IZ in mm
0
5
10
15
20
25
PEN G
OXA
CN
CIP
MXF
E
DA
LZD
TEC
VA
TE
TGC
FD
RD
SXT
Antibiotics

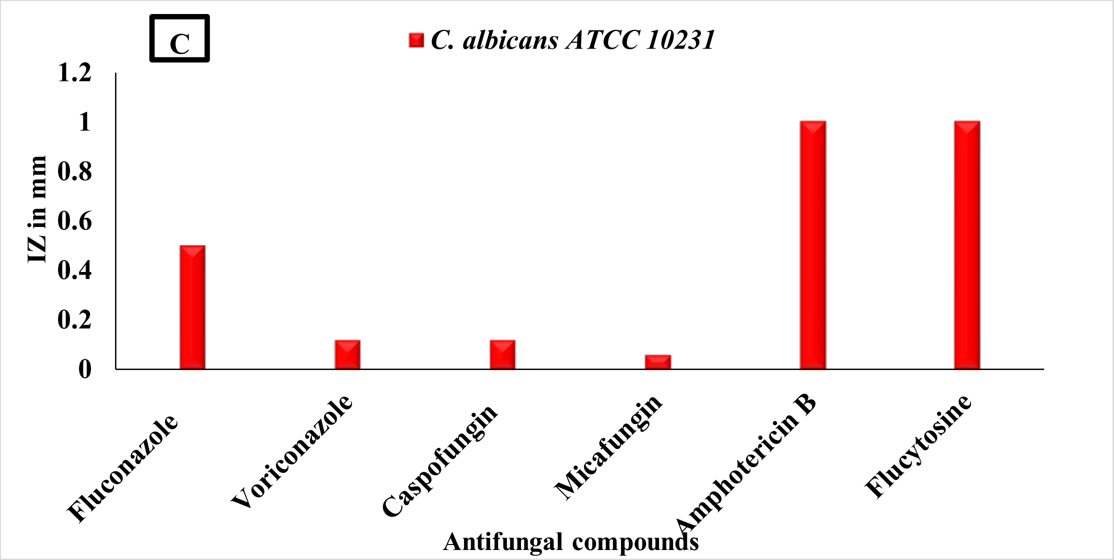

Figure (S3): Antimicrobial susceptibility of 15 antibiotic against **(a)** Gram negative bacterial strains and **(b)** Gram positive *Staphylococcus aureus* strain and antifungal susceptibility of about 6 antifungal compounds against **(c)** *Candida albicans*. Resistance profile was determined in inhibition zone (IZ).

Table (S1): **Chemical constituents of the ethyl acetate fraction of *Commiphora myrrha* resin obtained from GC/MS analysis**

Retention time	Compounds name	Molecular formula	Function	References
**11.807**	Cyclohexene, 4-ethenyl-4-methyl-3-(1-methylethenyl)1-(1-methylethyl)-, (3R-trans)-	C15H24	Antimicrobial	[1]
**14.852**	Cyclohexane, 1-ethenyl-1-methyl-2,4-bis (1-methylethenyl)-, [1S-(1. alpha, 2.beta, 4.beta.)]	C15H24	Antioxidant	[2]
**16.054**	1,5-Cyclodecadiene, 1,5-dimethyl-8-(1-methylethylidene	C15H24	Sesquiterpene (antimicrobial) compound known as Germacrene	[3]
**17.202**	Gamma-Muurolene	C15H24	Sesquiterpene (antioxidant and anti-inflammatory activities)	[4]
**19.275**	Selina-3,7(11)-diene	C15H24	Antioxidant	[5]
**21.491**	Benzofuran, 6-ethenyl-4,5,6,7-tetrahydro-3,6-dimethyl-5-isopropenyl-,	C15H20O	Natural product act as antioxidant	[6]

	trans-5-Isopropenyl-3,6-dimethyl-6-vinyl-4,5,6,7-tetrahydro-1-benzofuran			
**23.789**	Caryophyllene oxide	$C_{15}H_{24}O$	Natural sesquiterpene antimicrobial and antioxidant	[7]
**24.345**	Cyclopropene, 1-(3-acetoxy-1,1-dimethylhexan-5-onyl)-2-isopropenyl-	$C_{16}H_{24}O_3$	Antibacterial and antioxidant activities	[8]
**25.039**	3,7-Cyclodecadiene-1-methanol, alpha., alpha.,4,8-tetramethyl-, Hedycaryol 2-(4,8-Dimethyl-3,7-cyclodecadien-1-yl)-2-propanol	$C_{15}H_{26}O$	Natural product Antifungal, Antioxidant	[9]
**26.12**	Beta-Elemenone	$C_{15}H_{22}O$	Sesquiterpenoid (Anti-tumor)	[10]
**27.344**	Gamma-eudesmol	$C_{15}H_{26}O$	Sesquiterpenoid (exhibits antibacterial and antioxidant properties)	[11]
**27.582**	Isoledene	$C_{15}H_{24}$	Antimicrobial	[12]
**27.749**	3,7-Cyclodecadien-1-one, 3,7-dimethyl-10-(1-methylethylidene)-, Germacra-3,7(11),9-trien-6-one	$C_{15}H_{22}O$	Natural product Antifungal, Antioxidant	[13]
**27.97**	Selina-3,7(11)-diene	$C_{15}H_{24}$	Antioxidant	[14]
**28.181**	Bicyclo [3.1.1] hept-2-ene-2-ethanol, 6,6-dimethyl-, (1R)-2-(6,6 Dimethyl bicyclo[3.1.1]hept-2-en-2-yl)ethanol	$C_{11}H_{18}O$	Terpene antifungal	[15,16]
**28.706**	2-Naphthalenemethanol, 1,2,3,4,4a,5,6,8a-octahydro alpha, alpha.,4a,8-tetramethyl-, [2R-(2. alpha,4a. alpha.,8a. beta.)]-	$C_{15}H_{26}O$	Natural product sesquiterpenoid	[17]
**29.423**	Spathulenol	$C_{15}H_{24}O$	Sesquiterpenoid (anaesthetic and a vasodilator agent)	[18]
**29.752**	1-Naphthalenol, decahydro-1,4a-dimethyl-7-(1-methylethylidene)	$C_{15}H_{26}O$	Natural product Eudesm	[19]
**30.675**	Benzene, (1-cyclohexylethyl)-Ethane, 1-cyclohexyl-1-phenyl- (alpha-Methylbenzyl) cyclohexane 1-Phenyl-1-cyclohexylethane (1-Cyclohexylethyl	$C_{14}H_{20}$	Antioxidant and antibacterial	[20]

**31.556**	1H-Cycloprop[e]azulen-4-ol, decahydro-1,1,4,11	C15H26O	Natural product sesquiterpenes (anaesthetic and a vasodilator agent)	[21]
**32.552**	1-Heptatriacotanol	C37H76O	A fatty acid exhibited antioxidant and anticancer activities	[22]
**32.664**	4,7-Methanofuro[3,2-c] oxacycloundecin-6(4H-one, 7,8,9,12-tetrahydro-3,11-dimethyl-Germacra-1(10),7,11-trien-15-oic acid,8,12-epoxy-6-hydroxy	C15H18O3	Antioxidant and antibacterial	[23]
**33.153**	Nandrolone Acetate	C20H28O3	Steroid antioxidant	[24]
**33.477**	Aristolene epoxide	C15H24O	Monoterpenoid antioxidant	[25]
**37.831**	gamma-Elemene	C15H24	Sesquiterpene (anti-proliferative)	[26]
**39.775**	Ledene alcohol	C15H24O	Oxygenated sesquiterpenoids antioxidant	[27]
**39.915**	Shizukanolide	C15H18O2	Sesquiterpene moderate antifungal activity.	[28]
**43.3**	Eudesma-5,11(13)-dien-8,12-olide	C15H20O	Antioxidant and antibacterial	[29]

Table S2: Antibiotics used in this study

Antibiotics	Abbreviation
Penicillin G*	PEN G
Oxacillin*	OXA
Gentamicin	CN
Ciprofloxacin	CIP
Moxifloxacin	MXF
Erythromycin B*	E
Clindamycin*	DA
Linezolid*	LZD
Teicoplanin*	TEC
Vancomycin*	VA
Tetracycline	TE
Tigecycline	TGC
Fucidic acid*	FD
Rifampin*	RD
Trimethoprim/sulfamethoxazole	SXT
Ticarcillin	TIC
Ticarcillin/Clavulanic Acid	TIC/CLA
Piperacillin	PIP
Piperacillin/Tazobactam	TZP
Ceftazidime	CAZ
Cefepime	FEP
Aztreonam	ATM
Imipenem	IPM
Meropenem	MEM
Amikacin	AK
Tobramycin	TOB
Colistin	CST

Superscript (*) = Antibiotic used against *Staphylococcus* only and antibiotics without superscript letters = antibiotics used against both bacteria.

References

1 - Alanazi, N. A. H., Alamri, A. A., Mashlawi, A. M., Almuzaini, N., Mohamed, G., & Salama, S. A. Gas chromatography–mass spectrometry chemical profiling of commiphora myrrha resin extracts and evaluation of larvicidal, antioxidant, and cytotoxic activities. *Molecules*, **29**(8), 1778 (2024).

2- Salvi, P., Kumar, G., Gandass, N., Kajal, Verma, A., Rajarammohan, S., ... & Gautam, V.. Antimicrobial potential of essential oils from aromatic plant Ocimum sp.; a comparative biochemical profiling and in-silico analysis. *Agronomy*, **12**(3), 627 (2022).

3- Ahamad, S. R., Al-Ghadeer, A. R., Ali, R., Qamar, W., & Aljarboa, S. Analysis of inorganic and organic constituents of myrrh resin by gc–ms and icp-ms: an emphasis on medicinal assets. *Saudi Pharmaceutical Journal*, **25**(5), 788-794 (2017).

4- Martins, F. T., et al. Composition, and anti-inflammatory and antioxidant activities of the volatile oil from the fruit peel of Garcinia brasiliensis. *Chemistry & Biodiversity*, **5**(2), 251-258 (2008).

5- Menghini, L., et al. Chemical and bioinformatics analyses of the anti-leishmanial and anti-oxidant activities of hemp essential oil. *Biomolecules*, **11**(2), 272 (2021).

6- Kenchappa, R., Bodke, Y. D., Asha, B., Telkar, S., & Aruna Sindhe, M. Synthesis, antimicrobial, and antioxidant activity of benzofuran barbitone and benzofuran thiobarbitone derivatives. *Medicinal Chemistry Research*, **23**(6), 3065-3081 (2014).

7- Cascaes, M. M., et al. Essential oils from Annonaceae species from brazil: a systematic review of their phytochemistry, and biological activities. *International Journal of Molecular Sciences*, **22**(22), 12140 (2021).

8- Chen, D., Cheng, Y., Shi, L., Gao, X., Huang, Y., & Du, Z. Design, synthesis, and antimicrobial activity of amide derivatives containing cyclopropane. *Molecules*, **29**(17), 4124 (2024).

9- Roanisca, O., & Mahardika, R. G. Citrus x microcarpa bunge fruit extract as antibacterial against staphylococcus aureus. In *IOP conference series: earth and environmental science* (Vol. 599, No. 1, p. 012043). IOP Publishing (2020).

10- Feng, Y., et al. Beta-elemene: a phytochemical with promise as a drug candidate for tumor therapy and adjuvant tumor therapy. *Biomedicine & Pharmacotherapy*, **172**, 116266 (2024).

11- M'hamdi, Z., et al. Chemical composition and antibacterial activity of essential oil of Pelargonium graveolens and its fractions. *Arabian Journal of Chemistry*, **17**(1), 105375 (2024).

12- Mazimba, O., Masesane, I. B., Majinda, R. R., & Muzila, A. Gc-ms analysis and antimicrobial activities of the non-polar extracts of Mundulea sericea. *South African Journal of Chemistry*, **65**, 50-52 (2012).

13- Sharma, N., et al. Evaluation of the antifungal, antioxidant, and anti-diabetic potential of the essential oil of Curcuma longa leaves from the north-western himalayas by in vitro and in silico analysis. *Molecules*, **27**(22), 7664 (2022).

14- Agustini, S., et al. Phytochemical, gc-ms, and biological activity of extract of Pelawan tree (T. merguensis GRIFF.). *Rasayan Journal of Chemistry*, **16**(4) (2023).

15-Nikitina, L. E., et al. N-(((1 S, 5 R)-6, 6-Dimethylbicyclo [3.1. 1] hept-2-en-2-yl) methyl)-3-dodecan/tetradecanamido-N, N-dimethylpropan-1-aminium Bromide. *Molbank*, *2023*(3), M1704 (2023).

16- Qian, M., et al. Inhibitory mechanisms of promising antimicrobials from plant byproducts: A review. *Comprehensive Reviews in Food Science and Food Safety*, **22**(4), 2523-2590 (2023).

17- Mokaizh, A. A. B., Nour, A. H., & Kerboua, K. Ultrasonic-assisted extraction to enhance the recovery of bioactive phenolic compounds from Commiphora gileadensis leaves. *Ultrasonics Sonochemistry*, **105**, 106852 (2024).

18- de Souza Araújo, C., de Oliveira, A. P., Lima, R. N., Alves, P. B., Diniz, T. C., & da Silva Almeida, J. R. G. Chemical constituents and antioxidant activity of the essential oil from leaves of Annona vepretorum Mart.(Annonaceae). *Pharmacognosy Magazine*, **11**(43), 615 (2015).

19- Gautam, R. D., et al. Clonal propagation of Valeriana jatamansi retains the essential oil profile of mother plants: an approach toward generating homogenous grade of essential oil for industrial use. *Frontiers in Plant Science*, **12**, 738247 (2021).

20- Njateng, G. S. S., et al. Antidiabetic potential of methanol extracts from leaves of Piper umbellatum L. and Persea americana Mill. *Asian Pacific Journal of Tropical Biomedicine*, **8**(3), 160-165 (2018).

21-Alaboudi, K. A., et al. In vitro and in silico pharmacological effects of Rosmarinus officinalis leaf methanolic extracts and essential oils. *Scientific Reports*, **15**(1), 10699 (2025).

22-Salama, A., EL-Kassaby, M. I., Refaat, A., & Mohasib, R. M. Gc–ms and molecular docking analyses of phytochemicals from Calendula officinalis l. hexane extract and evaluation of its antioxidant and wound healing properties in rats. *Egyptian Journal of Chemistry*, **67**(13), 1037-1058 (2024).

23- Patrusheva, O. S., Ilyina, I. V., Salakhutdinov, N. F., Dragomanova, S. T., & Volcho, K. P. Synthesis of heterocyclic compounds with a cineole fragment in reactions of α-pinene-derived diol and monoterpenoid Aldehydes. *Compounds*, **5**(3), 25 (2025).

24-Câmara, L. C., & da Costa Viana, D. P. A structured review and critical analysis of RCTs on nandrolone decanoate's cardiac effects in young exercising man. *Journal of Pharmaceutical Research International*, **37**(3), 59-67 (2025).

25-Abraham, W. R., Riep, A., & Hanssen, H. P. Biotransformation and phylogeny: vi. microbial oxidation of aristolenepoxide to phytotoxins. *Bioorganic Chemistry*, **24**(1), 19-28 (1996).

26-Rana, M., et al. A standardized chemically modified Curcuma longa extract modulates irak-mapk signaling in inflammation and potentiates cytotoxicity. *Frontiers in Pharmacology*, **7**, 223 (2016).

27- Menichini, F., Conforti, F., Rigano, D., Formisano, C., Piozzi, F., & Senatore, F. Phytochemical composition, anti-inflammatory and antitumour activities of four Teucrium essential oils from greece. *Food Chemistry*, **115**(2), 679-686 (2009).

28-Wu, B., He, S., & Pan, Y. Sesquiterpenoid with new skeleton from Chloranthus henryi. *Tetrahedron letters*, **48**(3), 453-456 (2007).

29-Abdelwahab, S. I., et al. Phytochemical profiling of costus (Saussurea lappa Clarke) root essential oil, and its antimicrobial and toxicological effects. *Tropical Journal of Pharmaceutical Research*, **18**(10), 2155-2160 (2019).
